# Tailoring Genomic Selection for *Bos taurus indicus*: A Comprehensive Review of SNP Arrays and Reference Genomes

**DOI:** 10.3390/genes15121495

**Published:** 2024-11-21

**Authors:** Adebisi R. Ogunbawo, Henrique A. Mulim, Gabriel S. Campos, Allan P. Schinckel, Hinayah Rojas de Oliveira

**Affiliations:** 1 Department of Animal Sciences, Purdue University, West Lafayette, IN 47907, USA; aogunbaw@purdue.edu (A.R.O.); hmulim@purdue.edu (H.A.M.); gabrielsoarescampos@hotmail.com (G.S.C.); aschinck@purdue.edu (A.P.S.); 2 Department of Animal Biosciences, Interbull Centre, S-75007 Uppsala, Sweden

**Keywords:** breed-specific panels, genetic improvement, Zebu cattle

## Abstract

Background: Advances in SNP arrays and reference genome assemblies have significantly transformed cattle genomics, particularly for *Bos taurus indicus* (Zebu cattle). Many commercial SNP arrays were originally designed for *Bos taurus taurus*, leading to ascertainment bias and the exclusion of crucial SNPs specific to Zebu populations. This review assesses progress in SNP array and reference genome development, with a focus on efforts tailored to Zebu populations and their impact on genomic selection and breeding efficiency. Methods: We reviewed the relevant literature on the development of SNP arrays, reference genome assemblies, and SNP genotyping techniques used for Zebu cattle. Emphasis was placed on SNP arrays specifically designed for Zebu breeds, evaluating their contributions to genomic evaluations and identifying limitations in prediction accuracy. Results: Recent advancements, such as GeneSeek’s low- and high-density SNP panels, have aimed to reduce ascertainment bias and include key SNPs for Zebu populations by providing breed-specific panels. These panels have been instrumental in identifying genomic regions associated with economically important traits in Nellore cattle. Studies show that tailored SNP arrays and breed-specific reference genomes can enhance genetic diversity assessment and improve genomic predictions, supporting more effective breeding programs for Zebu cattle. Conclusions: Improved SNP arrays and breed-specific reference genomes are crucial for accurate genomic selection in Zebu cattle. Future efforts should prioritize expanding de novo *genome* assemblies, reducing ascertainment bias, and developing cost-effective genotyping solutions tailored to Zebu populations. Targeted genomic tools will ultimately enable more efficient breeding practices and enhance genomic selection for economically important traits in *B. t. indicus* cattle.

## 1. Introduction

Between 280,000 and 330,000 years ago, the now-extinct wild auroch (*Bos primegenius*) underwent significant genetic divergence, giving rise to two distinct groups, the humpless *B. t. taurus* (taurine) and the humped *B. t. indicus* (indicine or Zebu) [1,2,3]. While Zebu cattle are primarily distinguished by their hump, they also possess other notable traits, such as large ears, excess skin, and exceptional adaptability to harsh environments [1]. This adaptability has been further refined through historic selection for enhanced growth rates in challenging tropical conditions [4], following their domestication approximately 8000 years ago [1,5].

Traditionally, selection for breeding in livestock was primarily based on visual phenotypic observation. However, relying solely on these observable traits (phenotypes) without incorporating genetic or genomic data led to a relatively slow rate of genetic improvement [6]. Advances in quantitative genetics and animal breeding introduced the use of genetic relationships, such as pedigree information, to improve breeding decisions. This facilitated the development of the mixed models equations and best linear unbiased prediction (BLUP), which enabled a more accurate estimation of breeding values by considering the genetic relationships among individuals [7,8,9]. While pedigree-based selection improved accuracy compared to phenotype-only selection, the advent of genomic information has revolutionized breeding efficiency by further enhancing the precision of estimated breeding values and inbreeding coefficients [6].

Initially, traditional marker-assisted selection (MAS) used a limited number of genetic markers associated with specific traits of interest, combined with the pedigree information, to identify individuals with superior genetic potential. This approach was effective for traits controlled by a small number of genes, known as qualitative traits [10,11]. However, the efficiency of MAS was limited when dealing with complex or quantitative traits, which are influenced by the cumulative effects of thousands of genes and are highly influenced by the environment, typical of most economically important traits in livestock production [11]. To address these limitations, Meuwissen et al. [12] and Nejati-Javaremi et al. [13] conceptualized genomic selection, a method that uses a large number of genetic markers spread across an organism’s genome to predict breeding values without the need to identify the specific genes controlling the traits of interest [12]. Genomic selection became feasible in livestock populations with the discovery of single-nucleotide polymorphisms (SNPs). Unlike earlier genetic markers, such as restriction fragment length polymorphisms [14] and microsatellites [15], SNPs are abundant, easily detectable, and can be genotyped on a large scale using high-throughput technologies. Genomic selection assumes that SNP markers are in linkage disequilibrium with genes and/or quantitative trait loci (QTLs) influencing important traits. Moreover, the use of genomic information allows for the estimation of the realized relationship among individuals rather than relying solely on the average expected relationships from pedigree-based methods, which allows for a better understanding of the animal genetic resources.

The decreasing costs with genotyping using medium- and high-density SNP panels has significantly increased the number of genotyped animals, thereby enhancing the accuracy of genomic predictions. Although commercial SNP arrays and whole-genome sequences are available for studying Zebu cattle, these tools were primarily designed for *B. t. taurus*, which introduces potential bias when applied to *B. t. indicus*. For instance, the lower linkage disequilibrium (LD) in *B. t. indicus* compared to *B. t. taurus* [16] underscores the need for SNP panels specifically designed for *B. t. indicus* to improve the efficiency of genomic analyses, specifically by reducing the number of SNPs removed during quality control. Developing these tailored SNP panels can help not only mitigate bias but also increase the accuracy of genomic studies in Zebu cattle, ultimately supporting more effective breeding programs. A comprehensive review of advancements in SNP panels, particularly for *B. t. indicus*, is critical for assessing progress, identifying gaps, and guiding future efforts toward the creation of more efficient tools for genomic selection in Zebu populations.

## 2. The Rise of *B. t. indicus*

Domestic cattle can be broadly classified into two subspecies, namely *B. t. indicus* and *B. t. taurus*. These subspecies originated from a common ancestor, *Bos primigenius*, approximately 280,000 to 330,000 years ago [2,3,17]. *B. t. indicus* (Zebu) is characterized by distinct physical traits such as a fatty thoracic hump, large pendulous ears, and a prominent dewlap, which differentiate it from *B. t. taurus* [18]. The name “Zebu” is derived from the Tibetan words “Zen” or “Zeba”, meaning “hump of an animal” [18]. Zebu cattle have developed genetic and physiological adaptations that enable them to thrive in tropical climates. These adaptations include greater resistance to ectoparasites, tolerance to high temperatures and humidity, and the ability to consume low-quality forage. However, compared to *B. t. taurus*, Zebu cattle typically exhibit slower reproduction rates, later maturity, and delayed puberty [17,19,20,21,22].

**Figure 1:**
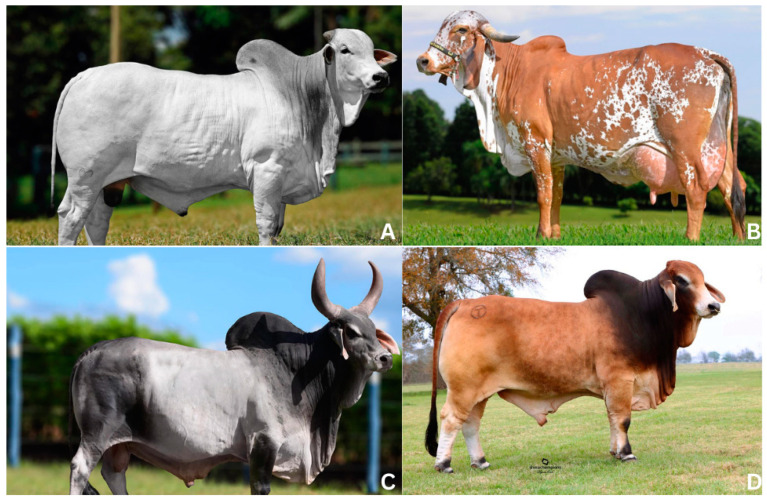
Main Zebu breeds used in breeding programs across the world. (**A**) Nellore animal, adapted from Alta Genetics Brazil (https://altagenetics.com.br, accessed on 25 October 2024); (**B**) Gyr animal, adapted from Almabari Agropecuaria (www.alambari.com, accessed on 25 October 2024); (**C**) Guzerat animal, adapted from Alta Genetics Brazil (https://altagenetics.com.br, accessed on 25 October 2024); (**D**) Brahman animal, adapted from Bovine Eline (https://www.bovine-elite.com, accessed on 25 October 2024).

Zebu cattle were domesticated approximately 10,000 years ago through selective breeding. The largest populations of Zebu cattle are currently found in India and Brazil, with around 302 million and 219 million animals, respectively [23]. The Nellore (beef cattle), Guzerat (dual-purpose cattle), and Gyr (dairy cattle) breeds, in particular, have significantly contributed to breeding programs of beef and dairy cattle worldwide [1,18,24,25] and have been instrumental in developing new breeds, such as Brahman [26] and Girolando [27]. Brahman cattle were established in the United States by crossbreeding native cattle with various *B. t. indicus* breeds from India [26]. Due to their adaptability to high temperatures, humidity, and resistance to parasites, along with their efficient use of high-fiber forages, Brahman cattle are well suited for production in temperate, subtropical, and tropical regions [26]. Genetic studies suggest that Brahman cattle are closely related to other zebuine breeds but carry notable taurine alleles, likely introduced through selective breeding and crossbreeding with local taurine breeds. Today, the Brahman breed is recognized for its valuable contributions to beef production despite generally lower reproductive rates than European breeds [26]. On the other hand, Girolando cattle were established in São Paulo, Brazil, and are a crossbreed between Gyr bulls and Holstein cows. This breed is known for its adaptability, precocity, and high milk production. Figure 1 shows the main Zebu breeds used in mating and breeding systems worldwide.

Zebu cattle are also highly valued in crossbreeding systems due to their resilience in hot climates and unique genetic characteristics, which contribute significantly to beef production [27]. Among the Zebu breeds, Nellore stands out in crossbreeding and breeding systems for beef production in tropical countries due to its adaptability, growth performance, and desirable genetic traits [28,29]. As the world’s largest beef exporter, Brazil relies heavily on Nellore cattle for its beef production, and the country hosts one of the largest Zebu populations globally [30]. Most of these cattle are traced back to animals imported from India, where Brazilian farmers from the Minas Gerais state traveled to North India between 1913 and 1917 to acquire Zebu bulls and cows. This initiative was part of a large movement to standardize cattle breeds and industrialize breeding practices in Brazil [31]. Over time, Brazil developed its own breeding programs tailored to national and market needs. Today, numerous independent Nellore breeding programs manage over half a million cattle annually in Brazil, using both traditional pedigree-based and genomic selection. Prominent programs include the Brazilian Association of Zebu Breeders (ABCZ), CIA do Melhoramento, DeltaGen, Nelore Qualitas, PAINT, Agropecuária Jacarezinho, and Aliança Nelore [23,32,33,34]. Nellore is also widely utilized in crossbreeding initiatives designed to create synthetic breeds adapted to tropical environments [35]. After a period of decline, crossbreeding programs in Brazil saw renewed focus in the early 1970s, with Nellore cattle playing a central role in these revitalized efforts [36].

## 3. DNA and the Development of Molecular Markers

Deoxyribonucleic acid (DNA) is a double-stranded helical molecule composed of purine and pyrimidine bases linked by a phosphate backbone and hydrogen bonds [37,38]. As the carrier of genetic information, DNA is replicated during cell division, controlling the transmission of alleles from one generation to the next [35]. The specific pairing of bases is fundamental for the formation of the DNA structure; adenine (a purine) bonds exclusively with thymine (a pyrimidine), while guanine (another purine) pairs with cytosine (a pyrimidine) [37]. Although the proportion of bases may vary among species, the molar ratios between them remain relatively constant, typically close to one [37,39]. Notably, it has been discovered that between 30% and 90% of the genome comprises repetitive DNA segments that exhibit polymorphisms [40].

Molecular markers, also known as genetic markers, are important tools in animal breeding, helping to determine an animal’s genetic composition and predict its performance with greater accuracy [41,42]. The identification and selection of specific DNA polymorphisms using molecular markers represent significant advancements in molecular genetics [43]. Molecular markers can be broadly classified as hybridization-based markers (RFLP), polymerase chain reaction (PCR)-based markers (RAPD, AFLP, microsatellites), and DNA chip and sequencing-based markers (SNPs). Below, we provide a brief overview of the main characteristics and differences between these types of molecular markers used in animal breeding, arranged chronologically by their discovery. For a complete description of these methods, please refer to Singh et al. [41], Reshma and Das [42], and Dhutmal et al. [43].

### 3.1. Hybridization-Based Markers

Hybridization-based markers enable the visualization of DNA profiles by hybridizing DNA, previously digested with restriction enzymes, to a labeled probe. This probe can be a DNA fragment with a known sequence or origin [41,43,44]. A notable example of hybridization-based markers is the restriction fragment length polymorphism (RFLP).

#### Restriction Fragment Length Polymorphism (RFLP)

The inception of DNA marker technology can be traced back to the creation of the first molecular map of the human genome [45]. This groundbreaking achievement introduced RFLP as a foundational tool for subsequent advancements in genomics and the development of more sophisticated molecular markers [45]. RFLP involves digesting DNA with restriction enzymes, producing fragments of varying lengths. These fragments are then hybridized with probes labeled with radioactive material, enabling visualization through autoradiography. RFLPs are highly informative and reliable, allowing for the differentiation of various genotypes, the identification of distinct loci, and the use of co-dominant markers [45]. However, this technique has its limitations, including difficulties in distinguishing similar alleles in closely related species due to high-molecular-weight DNA requirements, labor-intensive staining methods, and restricted applicability in genetic mapping [46]. These challenges have led to the development of alternative technologies, such as polymerase chain reaction (PCR)-based techniques, to overcome these constraints [46].

The first practical application of RFLPs was in pigs, where human leucocyte antigen cDNA probes were employed to examine the RFLP of the major histocompatibility complex, known as swine leukocyte antigen [47]. The effectiveness of RFLPs in illustrating genetic diversity within dairy cattle populations was validated through studies of Israeli Holstein Friesian dairy bulls [48]. These bulls were screened for RFLPs by hybridizing cloned DNA probes specific to bovine growth hormone, chymosin, and rat muscle β-actin with bovine DNA [48]. The identification of RFLPs has ushered in a new era for studying intraspecies variation [49].

### 3.2. Polymerase Chain Reaction-Based Markers

Polymerase chain reaction-based markers, also known as amplification markers, revolutionized genetic analysis by enabling the in vitro amplification of specific DNA segments. This technique allows researchers to visualize amplified DNA as distinct bands on a gel, providing a straightforward method for detecting genetic variation. Prominent examples of PCR-based markers include random amplified polymorphic DNA (RAPD), amplified fragment length polymorphisms (AFLPs), and simple sequence repeat markers (SSR), commonly known as microsatellites [41,43].

#### 3.2.1. Random Amplified Polymorphic DNA (RAPD)

Random amplified polymorphic DNA assays are non-radioactive methods used to detect polymorphisms at multiple loci [50]. These assays require only a small amount of DNA and less technical expertise, making them more accessible. The identification of RAPD amplicons is achieved through bulked segregant analysis [50]. However, RAPD’s usefulness in molecular mapping is limited by the appearance of non-parental bands in the progeny [51]. Additionally, RAPD suffers from poor reproducibility due to low annealing temperatures, leading to mispairing [52]. To improve reproducibility, strict adherence to experimental protocols is recommended [53].

The first use of RAPD was in *Arabidopsis thaliana*, a model organism in plant molecular genetics, due to its compact genome and short generation time [54]. In this study, RAPD was used to create a high-density genetic map with 252 markers, integrated into a recombinant inbred population of *A. thaliana*, showing how RAPD could rapidly saturate genetic maps [54,55].

#### 3.2.2. Amplified Fragment Length Polymorphism (AFLP)

Amplified fragment length polymorphism is a PCR-based method that combines restriction enzymes with oligonucleotide adapters to selectively amplify restriction fragments [56]. These fragments are then separated on polyacrylamide sequencing gels, with polymorphisms detected through mutations in the restriction recognition sites [46]. The AFLP method is highly reproducible, fast, and generates a high frequency of polymorphisms, making it particularly effective for linkage analysis in segregating populations [44] and constructing genetic linkage maps [57]. However, AFLP has limitations, including lower polymorphic information content compared to microsatellites and higher costs associated with labor-intensive protocols [46]. In a study comparing the levels of polymorphism among AFLP, RAPD, and microsatellites in 12 japonica and two indica rice varieties, AFLP demonstrated a higher number of polymorphic bands compared to the other markers, highlighting its potential in mapping important genes using versatile primer combinations [58].

#### 3.2.3. Simple Sequence Repeat Markers (SSR) or Microsatellites

Microsatellites, also known as simple sequence repeat (SSR) markers, are short repetitive sequences found abundantly throughout the genome. They consist of motifs made up of 1–6 nucleotide bases that are repeated in tandem. These motifs often vary in the number of repeats due to imprecise replication during DNA synthesis, contributing to their polymorphism [46]. SSR markers are highly reliable, exhibit a higher level of polymorphism compared to other molecular markers, and were widely used for genetic analysis in the 1980s [59]. However, developing microsatellite markers can be complex and resource-intensive, involving tasks such as the construction of genomic libraries, sequencing clones, designing primers, and testing for polymorphisms. This process is both time-consuming and costly. To address these challenges, researchers often outsource these tasks to external services or use software tools such as Msatcommander, E-TRA, and Msatfinder for microsatellite mining [60,61].

In cattle, microsatellites were initially used for animal identification and pedigree verification [62]. These markers established a reliable panel for parentage testing in cattle populations, which is essential for implementing efficient breeding programs. The accuracy of microsatellite-based pedigree testing stems from the direct relationship between marker polymorphism and the ability to detect errors in parentage assignments [62].

### 3.3. Sequencing-Based Markers

Sequencing-based markers represent a powerful tool in genomic analysis, leveraging advancements in DNA sequencing technology to detect and analyze variations at the nucleotide level across genomes. Unlike hybridization and PCR-based markers, which focus on detecting specific DNA regions or amplifying target sequences, sequencing-based markers allow for a comprehensive examination of genetic diversity across entire genomes [44]. This approach has enabled the identification of a wide range of genetic variants, from single-nucleotide changes to structural variations, with applications in population genetics, evolutionary studies, and genome-wide association studies (GWASs). A prominent example within sequencing-based markers is the single-nucleotide polymorphism (SNP).

#### Single-Nucleotide Polymorphisms (SNPs)

Single-nucleotide polymorphisms (SNPs) are defined as variations at a single nucleotide position in the genome that occur in at least 1% of a population [63]. As the most common form of genetic variation, SNPs are extensively used to study genetic differences among individuals and populations. The number of identified SNPs varies significantly between species and even among breeds within the same species. For example, the 1000 Bull Genomes Project identified 28.3 million genetic variants, including 26.7 million SNPs, through the whole-genome sequencing (WGS) of 234 Holstein cattle [64]. More recently, Sun et al. [65] reported the discovery of 15,154,539 autosomal SNPs in Simmental cattle.

Earlier studies provided substantial SNP discoveries; Eck et al. [66] identified 2.44 million SNPs in Hereford cattle using next-generation sequencing, with 82% of these SNPs being previously unreported and subsequently added to public SNP databases. Similarly, Weldenegodguad et al. [67] identified 17.45 million SNPs across 15 cattle breeds, with the Yakutian breed showing the highest SNP count. Even before the widespread adoption of WGS, the Bovine HapMap Consortium developed probes from a Hereford female and six other breeds, enabling the assessment of 37,470 SNPs across 497 cattle from 19 geographically and biologically diverse breeds [68].

Further work by Van Tassell et al. [69] added 23,000 SNPs to the bovine SNP database by studying 66 cattle from various breeds, including Holstein, Angus, Red Angus, Gelbvieh, Hereford, Limousin, and Simmental. This effort culminated in the creation of a 54,001-SNP array, a commercially available tool for genomic analysis that enables the simultaneous genotyping of thousands of SNPs across the genome at a reduced cost [70,71,72].

## 4. History of SNP Arrays

Affymetrix and Illumina, both pivotal in human and animal genetics, are the primary developers of SNP arrays used for genomic analyses across various species, including livestock [73,74]. Affymetrix pioneered high-throughput SNP genotyping with microarray technology in the early 1990s, initially focusing on human health applications but later expanding into livestock applications with arrays designed for cattle, swine, poultry, and other animals [60,61]. During this period, Affymetrix introduced the first commercial high-density and high-throughput SNP genotyping array. However, this initial array, which supported genotyping for only 2000 SNPs, was insufficient for comprehensive GWASs and genomic predictions [75]. Later, Affymetrix developed the Affy10K cattle genotyping array to assist in linkage mapping and parentage verification, utilizing the Bovine Mapping 10K SNPs identified during the bovine genome sequencing project [76,77]. However, the sparse SNP density—just 1.71 SNPs per centimorgan—limited its broader applicability [75,78].

To address these challenges, Professor David Walt and his team at Tufts University collaborated with Illumina to develop bead array technology [79]. This innovative technology utilized silica beads coated with DNA probes, which hybridize with complementary DNA in genomic samples, providing an efficient and cost-effective method for high-density SNP genotyping [78]. In January 2010, Illumina introduced the BovineHD Genotyping BeadChip, a high-density array containing 777,962 SNPs [72]. Shortly after, in 2011, Affymetrix responded by releasing the Axiom Genome-Wide BOS 1 Array, containing 648,874 SNPs [73]. Despite these increases in SNP density, these arrays did not substantially enhance the accuracy of genomic predictions [12]. Additionally, their higher costs compared to low- and medium-density SNP panels, along with the greater computational demands for analyzing the high-density data, still limit their widespread use for routine genomic evaluations [79,80].

Consequently, in September 2010, Illumina launched the GoldenGate Bovine3K BeadChip, which featured 2900 SNPs. This array was designed to provide affordable genotyping for cattle producers using GoldenGate assay chemistry. Although this SNP panel offered a significantly smaller number of SNPs compared to the other panels, it provided a cost-effective solution for parentage validation and cattle traceability [81]. By September 2011, Illumina introduced the Bovine Low-Density (LD) chip, containing 6909 SNPs. This array offered high imputation accuracy at a reduced cost [76], making it ideal for deriving genomic estimated breeding values (GEBVs) from imputed genotype data. Unlike the Bovine3K chip, the Bovine LD chip could be customized with additional SNPs and used the same chemistry as the BovineSNP50 chip [75,76,81].

Leveraging the SNP arrays developed by Illumina and Affimetrix, companies like Neogen (formerly GeneSeek; Lincoln, NE, USA) and Zoetis (Parsippany, NJ, USA) have become major commercial providers of genotyping services for livestock, particularly cattle. Neogen and Zoetis offer affordable, large-scale SNP genotyping tailored to meet the needs of the livestock industry, using Illumina and Affymetrix panels adapted specifically for their needs. These commercial services have significantly contributed to advancing genetic selection, parentage testing, and GWASs in cattle and other livestock populations, making genomic tools accessible for routine breeding applications and genetic evaluations [60]. A summary of key SNP arrays developed for cattle over the years is shown in Table 1.

The introduction of the first bovine SNP array marked a pivotal moment in advancement in SNP array technology, benefiting not only cattle but also other livestock species. Its cost-effectiveness, particularly for genotyping large sample sizes, positioned it as a superior alternative to traditional methods [91]. The significant differences in linkage disequilibrium between *B. t. taurus* and *B. t. indicus* drove the development of specialized SNP arrays tailored for Zebu cattle, addressing the specific genetic needs of these breeds [16].

### SNP Arrays Specific to Zebu Cattle

While several commercial SNP arrays are available for cattle research, it is important to note that these genomic tools were initially designed for *B. t. taurus*. As a result, many SNPs are often removed during the quality control step due to their fixation within the Zebu populations [92]. Additionally, some key SNPs that are relevant to Zebu cattle may not be included in these panels. This omission can introduce biases in diversity studies, reduce the accuracy of genomic predictions, and hinder the identification of critical QTLs and genes in GWASs [1,27,93,94]. For instance, most SNP arrays developed by Illumina and Affymetrix were developed for taurine breeds (e.g., Angus, Simmental, Jersey, Holstein, and Hereford) and thus may not effectively capture breed-specific or informative genetic variants for Zebu cattle [72,95].

To overcome these limitations, SNP arrays specifically designed for *B. t. indicus* have been developed. For instance, in February 2012, GeneSeek (current Neogen) introduced the GeneSeek Genomic Profiler (GGP) Indicus-specific array (Table 1) [96]. Following this, GeneSeek released several other arrays, including the SGGP-20Ki (a low-density SNP panel with approximately 18,860 SNPs), the GGP-75Ki (a high-density SNP panel with approximately 74,677 SNPs), and the GGP-HDi, which also contains approximately 74,677 SNPs (Table 1) [97,98]. These SNP arrays were designed to address the ascertainment bias observed with taurine-focused chips like the Illumina BovineSNP50 chip [85]. Nayee et al. [97] also demonstrated that the GGP-75Ki is a better choice for genotyping *B. t. indicus* animals compared to the BovineSNP50 chip due to its higher number of polymorphic SNPs. Similarly, Zoetis also developed a breed-specific targeted SNP panel known as the Clarifide Nelore^®^ panel, along with tailored genomic predictions for Nellore cattle [90]. The Clarifide Nelore^®^ panel has proven instrumental in identifying key genomic regions associated with economically significant traits in Nellore cattle.

## 5. Reference Genome Assemblies

The development of cattle reference genome assemblies has progressed significantly, driven by advancements in sequencing technologies and assembly methodologies. Early assemblies, such as UMD3.1 and Btau4.1, were constructed using clone-by-clone and whole-genome shotgun sequencing methods. Although pioneering, these assemblies had substantial limitations, including assembly gaps, misassembles, and errors, particularly in complex genomic regions undergoing rearrangements in somatic cells [75,99]. With the introduction of ARS-UCD1.2, built from the same Hereford individual as previous assemblies, more sophisticated technologies were used to yield a highly continuous, accurate, and complete reference genome [100]. This assembly spans 2.7 Gb and is over 250 times more continuous than earlier versions, with notably improved gene annotation that enables more precise genetic studies and trait analysis across the species [75,100].

To better capture the genetic diversity within cattle, recent efforts have focused on developing breed-specific assemblies and a cattle pangenome. The pangenome incorporates multiple high-quality assemblies from various breeds, ensuring a broader representation of genetic variation. Breed-specific assemblies, such as ARS_Simm1.0 for Simmental cattle and references for African breeds, improve the inclusivity of global cattle diversity in genomic analyses [101,102]. Studies have demonstrated that the choice of reference genome, such as ARS-UCD1.2 or UOA_Angus_1, significantly impacts the accuracy of read mapping and variant genotyping, underscoring the importance of selecting appropriate references for different breeds [103]. Emerging technologies, including long-read sequencing and trio binning, promise to deliver fully haplotype-resolved genomes, further advancing cattle genomics and supporting future research aimed at improving cattle genetics [75,104].

### Reference Genome Assemblies for Zebu Cattle

In February 2012, the first reference genome for *B. t. indicus* was published, based on a Nellore animal sequenced at 52X coverage using the SOLiD sequencing platform [95,105]. Notably, this pioneering reference genome was not created through de novo assembly but by aligning reads to the *B. t. taurus* reference genome (BTAU version 4.0) [95,105]. In February 2018, a second reference genome for *B. t. indicus*, based on a Gyr animal, was released using a combination of sequencing platforms, including 454, Ion Torrent, and Illumina (NextSeq and MiSeq). Like the previous version, this genome relied on read mapping to the *B. t. taurus* UMD version 3.1.1 reference rather than de novo assembly [99]. The first de novo assembly for *B. t. indicus* was created using high-coverage Illumina short reads for a Nellore bull named Futuro and assembled with the DeNovoMAGIC 3.0 NRGene pipeline. Chromosomal sequences were constructed with the aid of linkage disequilibrium data from over 2000 Nellore animals genotyped with more than 777,000 SNP markers [17]. This assembly allowed for a more accurate evaluation of key haplotype blocks, improving assessments of genetic diversity in Nellore and other *B. t. indicus* breeds [95,106].

## 6. Studies Using SNP Arrays and/or Genome Assemblies Specific to Zebu Cattle

New SNP arrays specifically designed for *B. t. indicus* have been developed over time. Genotyping *B. t. indicus* cattle using SNP arrays designed for *B. t. taurus* often resulted in artificially high levels of genome homozygosity [17]. The widely used Illumina Bovine SNP50 array was insufficient for accurate genomic predictions and selection in *Bos indicus* cattle due to the significant genetic differences between the two subspecies. One of the first SNP panels customized for *B. t. indicus* was the Clarifide Nellore 2.0, featuring around 12,000 SNP markers. This panel was used in an early study to identify genomic regions associated with carcass traits, with genotypic data from 8652 animals. These low-density data were then imputed to a higher density using the Illumina BovineHD BeadChip [107]. Later, the Clarifide Nellore 3.1 panel, containing over 29,001 SNPs, was employed in a study of calving ease in 1201 Nellore heifers, with a similar imputation to higher density [108].

The Clarifide Nellore panel has been paramount for various research initiatives. For instance, Stafuzza et al. [109] and Kluska et al. [110] used the Clarifide Nellore 2.0 low-density panel (12,000 SNPs) to genotype over 1365 and 3331 Nellore cattle, respectively, for studies on the hornless trait and estimates of heritability and genetic correlations within the population. These studies also imputed data to a higher density [109,110]. Further research by Carvalho et al. [111] used the Clarifide Nellore 3.1 panel to investigate genomic regions influencing growth traits in 7689 Nellore cattle. Likewise, Burnes et al. [112] employed the same panel to genotype Nellore cattle using DNA from hair follicles, improving assessments of challenging traits such as feed efficiency (Burnes et al., 2020). Across these studies, the use of low-density SNP panels, followed by imputation to a higher density, proved effective for accurate genomic predictions in *B. t. indicus* breeds, highlighting the value of customized SNP arrays for advancing genomic selection in Zebu cattle.

Another major advancement came in February 2012 with the launch of the GeneSeek Genomic Profiler (GGP) Indicus-specific array, based on the Illumina BovineLD genotyping BeadChip [96]. Subsequent studies confirmed that the Illumina BovineHD and BovineSNP50 chips, initially developed for *B. t. taurus*, exhibited higher levels of monomorphism in *B. t. indicus* cattle, making them less suitable for genotyping these breeds [97]. In contrast, the GeneSeek 75K Indicus Chip (GGP-75Ki), containing around 75,000 SNPs, proved far more suitable for *B. indicus* genotyping [97]. The GeneSeek Super Genomic Profiler Indicus (SGGP-20Ki) is a low-density panel containing approximately 18,860 SNPs. This cost-effective array has been widely used in genomic evaluations of *B. indicus* populations, with animals genotyped using the SGGP-20Ki panel and imputed to higher-density arrays to reduce costs while maintaining accuracy [85,86,113,114]. For more detailed genomic analysis, the high-density GeneSeek Genomic Profiler Indicus (GGP-75Ki) array, containing around 74,677 SNPs, is available. This panel is typically used for reference animals, while selection candidates are genotyped using a lower-density panel followed by imputation to higher densities [85,86].

Regarding the impact on genetic diversity, Mulim et al. [115], in their study across various breeds—including *B. t. indicus* breeds such as Nellore (genotyped with different densities of the GGP), Brahman, and Gyr—found that the choice of the genotyping panel can significantly impact the results of certain genetic diversity parameters. In this context, they observed that using a panel designed specifically for *B. t. indicus* breeds (such as the GGP indicus 35K SNP panel), which includes SNPs selected for higher minor allele frequency in Zebu populations and ensures optimized equidistant marker spacing, could influence the detection of parameters like runs of homozygosity (ROH). This finding aligns with Chen et al. [116], who analyzed global genetic diversity, introgression, and evolutionary adaptations in indicine cattle using whole-genome sequencing. Their research identified a total of 67,162,108 autosomal SNPs across various indicine and taurine populations, revealing that genome-wide nucleotide diversity was generally higher in indicine cattle compared to taurine cattle. Additionally, they also found lower genetic distances among indicine breeds compared to the genetic distances between indicine and taurine groups, indicating a closer genetic relationship within indicine breeds.

In conclusion, using a *B. t. taurus* reference genome to genotype indicine cattle can lead to biases. Due to the differences observed between the *B. t. taurus* and *B. t. indicus* genomes, using a reference genome created using *B. t. taurus* animals may result in the underrepresentation of SNPs and other genetic variants specific to Zebu cattle. This underrepresentation can obscure certain genetic traits, especially those related to morphology, immune response, disease resistance, and heat tolerance, which are paramount for adapting these breeds to global climate change [116]. Consequently, genotyping Zebu cattle with a taurine-based platform may overlook critical adaptations, limiting insights into their evolutionary history and breeding strategies.

To better capture genetic diversity in cattle, recent efforts have focused on developing breed-specific assemblies and a comprehensive cattle pangenome. This pangenome integrates multiple high-quality genome assemblies from various breeds to more accurately represent cattle genetic variation. Led by the Bovine Pangenome Consortium (BPC), an international collaboration, this initiative aims to build a more complete picture of cattle genomic diversity [117]. A significant step has been made with the creation of a haplotype-resolved assembly for the Brahman breed [118] inside the Zebu cattle scenario; however, including additional breeds is essential to capture the full diversity within cattle.

Expanding the pangenome to incorporate more Zebu cattle breeds is especially valuable for understanding the unique genetic diversity and traits of this subspecies, which are crucial for breeding programs and conservation efforts. Reference-quality genome assemblies for Zebu cattle would result in a more comprehensive and representative reference genome, improving the accuracy of genomic evaluations and predictions for key production traits, which is vital for optimizing breeding strategies.

The BPC is also working to establish a prioritized list of breeds and species for genome assembly projects, which ensures resources are allocated efficiently and the genetic characteristics of Zebu cattle are well-represented in genomic studies [117]. This approach supports conservation efforts, particularly in regions where Zebu cattle play a vital role in agriculture and local livelihoods. Documenting the genetic composition of Zebu cattle can help preserve unique traits that may enhance their adaptation to environmental changes. By including more Zebu cattle in the program, genomics research can benefit regions where large-scale genotyping is less accessible, ultimately leading to improved breeding practices and productivity.

## 7. Future Directions and Limitations in SNP Genotyping for Zebu Cattle

Future research in SNP genotyping for Zebu cattle must focus on capturing the unique genetic diversity of *B. t. indicus* breeds. While significant advances in SNP array development have been made, limitations remain that impact the accuracy and effectiveness of genomic selection. Custom SNP panels that reflect Zebu-specific genetic variants will likely improve the accuracy of selection for economically valuable traits in Zebu cattle, such as fertility, feed efficiency, and disease resistance. Furthermore, addressing ascertainment bias is also essential, as many SNP arrays were initially based on *B. t. taurus* populations, which limits their application for Zebu cattle. Incorporating SNPs identified directly from Zebu populations can mitigate this bias and boost prediction accuracy for complex traits. Zebu-specific panels can also be used to target genetic traits and variations within composite breeds, thereby capturing additive and dominance effects more effectively within these populations. This approach enables the identification of genes that may segregate uniquely within one population or another, as well as the discovery of SNPs associated with particularly desirable genes that are actively segregating in the population.

While breed-specific SNP panels can make genomic selection more efficient, whole-genome sequencing (WGS) offers a flexible and powerful alternative that allows researchers to directly select the most relevant markers for specific analyses. WGS enables the direct identification of causal mutations, effectively capturing breed-specific genetic diversity without reliance on standardized SNP panels. Therefore, by incorporating WGS, researchers can overcome ascertainment bias associated with SNP arrays developed for *B. t. taurus* breeds, moving beyond “one-size-fits-all” approaches. However, reducing costs remains essential for a broader implementation of genomic tools in Zebu breeding programs. Advances in affordable methods, such as low-pass sequencing (LPS), provide a cost-effective solution while maintaining high data quality, making genomic evaluations more accessible. Collectively, innovations in WGS, breed-specific SNP panels, and affordable genotyping options promise to enhance genomic selection in Zebu cattle, maximizing genetic improvements tailored to diverse indicine populations.

## Figures and Tables

**Table 1:** Summary of key SNP arrays developed over the years.

Genotyping Arrays	Label	Genotyping Technology	Number of SNPs	Genotyping Company	Year	Reference
GeneChip Bovine Mapping 10K	Affy 10K	Synthetic oligonucleotide microarrays	9919	Affymetrix	2004	[75]
GeneChip Bovine Mapping 25k	25K	Molecular inversion probe	25,000	Affymetrix	2007	[82,83]
Illumina Bovine SNP50	50K	Infinium high-throughput screening	54,001	Illumina	2007	[76,84]
GoldenGate Bovine 3K	3K	GoldenGate assay chemistry	2900	Illumina	2010	[76,77]
Illumina Bovine HD Genotyping	HD	Infinium HD assay	777,962	Illumina	2010	[76]
Illumina Bovine Low Density	7K	Infinium high-throughput screening	6909	Illumina	2011	[81]
Axiom Genome-Wide BOS 1 Array	BOS 1	Axiom genotyping assay	648,874	Affymetrix	2011	[73]
Geneseek Genomic Profiler (GGP)	GGP	Illumina Bovine LD genotyping beadchip	8654	Neogen ^1^	2012	[76]
Geneseek 75k indicus chip	GGP 75Ki	Illumina platform	74,677	Neogen ^1^	2013 ^4^	[85,86]
Genemax Advantage	GMX	Illumina platform	~50,000	Zoetis and AGInc ^2^	2016	[87]
CLARIFIDE Plus	50k	Illumina platform	45,245	Zoetis ^3^	2016	[88]
GGP indicus 35k micro array	GGP 35Ki	Illumina platform	35,339	Neogen ^1^	2017 ^4^	[89]
CLARIFIDE Nelore	ZBU	Illumina platform	~12,000	Zoetis ^3^	2018	[90]

Commercial SNP panels developed by Illumina specifically for ^1^ Neogen Corporation, ^2^ Zoetis and Angus Genetics Inc., and ^3^ Zoetis. ^4^ Personal communication.

## Data Availability

Not applicable.
